# Automatic diagnosis and classification of breast surgical samples with dynamic full-field OCT and machine learning

**DOI:** 10.1117/1.JMI.10.3.034504

**Published:** 2023-06-01

**Authors:** Jules Scholler, Diana Mandache, Marie Christine Mathieu, Aïcha Ben Lakhdar, Marie Darche, Tual Monfort, Claude Boccara, Jean-Christophe Olivo-Marin, Kate Grieve, Vannary Meas-Yedid, Emilie Benoit a la Guillaume, Olivier Thouvenin

**Affiliations:** a PSL University, Institut Langevin, ESPCI Paris, CNRS, Paris, France; b AQUYRE Bioscences-LLTech SAS, Paris, France; c Institut Pasteur, Bioimage Analysis Unit, Paris, France; d Gustave Roussy Cancer Campus, Department of Medical Biology and Pathology, Villejuif, France; e SCM Bichat, Paris, France; f Sorbonne Université, Institut de la Vision, INSERM, CNRS, Paris, France; g Quinze-Vingts National Eye Hospital, Paris, France

**Keywords:** label-free histopathology, dynamic optical coherence tomography, metabolic imaging, machine learning, automated diagnosis

## Abstract

**Purpose:**

The adoption of emerging imaging technologies in the medical community is often hampered when they provide a new unfamiliar contrast that requires experience to be interpreted. Dynamic full-field optical coherence tomography (D-FF-OCT) microscopy is such an emerging technique. It provides fast, high-resolution images of excised tissues with a contrast comparable to H&E histology but without any tissue preparation and alteration.

**Approach:**

We designed and compared two machine learning approaches to support interpretation of D-FF-OCT images of breast surgical specimens and thus provide tools to facilitate medical adoption. We conducted a pilot study on 51 breast lumpectomy and mastectomy surgical specimens and more than 1000 individual $1.3\times 1.3\;{\mathrm{mm}}^{2}$ images and compared with standard H&E histology diagnosis.

**Results:**

Using our automatic diagnosis algorithms, we obtained an accuracy above 88% at the image level ($1.3\times 1.3\;{\mathrm{mm}}^{2}$) and above 96% at the specimen level (above ${\mathrm{cm}}^{2}$).

**Conclusions:**

Altogether, these results demonstrate the high potential of D-FF-OCT coupled to machine learning to provide a rapid, automatic, and accurate histopathology diagnosis with minimal sample alteration.

## 1 Introduction

In 2018, cancer was the most frequent cause of premature death in 48 countries in the world.^1^ Breast cancer was the most frequently diagnosed cancer among females in 154 countries.^1^ When early diagnosis is made, conservative surgery is preferred to partially preserve organ function and maintain the patient’s body image and quality of life.^2^^,^^3^ Nonetheless, these surgeries could be associated with higher risk of recurrence, risk of re-excision, and potential delay to the initiation of other therapies.^3^^,^^4^ The difficulty partly lies in the efficient evaluation of tumor margins directly during surgery. Techniques such as intraoperative touch-preparation cytology or frozen-section analysis can be performed during the surgery and have shown promising potential in reducing the re-excision rate after primary breast conserving surgery by a factor 3 compared with permanent section histopathology.^5^ Nonetheless, these techniques still require about 30 min during the surgery, are resource intensive, are associated with various artifacts (sampling, freezing artifacts), and offer a less precise diagnosis compared with standard histopathological assessment.^6^^–^^8^ A recent review compared frozen sections and touch-preparation cytology to regular histology on 4300 and 1900 breast cancer cases and reported a sensitivity/specificity of 0.86/0.96 for frozen sections and 0.91/0.95 for cytology.^8^ This review also pointed out that these intraoperative margin assessment techniques are not commonly routinely used in hospitals. Thus, improving intraoperative diagnosis toward a real-time, easily applicable process combining large-scale assessment and cell resolution measurement to accurately evaluate cancer margins is an important step toward reducing the re-excision rate, the risk of recurrence, as well as patient stress and use of medical resources.

It has been the reason for the development of many optical digital techniques over the past few years. Among others, quantitative phase imaging,^9^ optical coherence tomography (OCT),^10^^–^^12^ Raman spectroscopy,^13^^,^^14^ fluorescence lifetime imaging,^15^^,^^16^ optical elastography,^17^ and multiphoton microscopies^18^^,^^19^ showed high potential to offer an accurate histopathological diagnosis. Nonetheless, none of these techniques have emerged as a gold standard, and they all provide slightly different contrasts that often do not directly compare to the gold standard hematoxylin–eosin (H&E) histology. As a consequence, these promising techniques are hardly adopted by the medical community since adapting to a new contrast is challenging and time consuming, and such efforts are not necessarily transferable from one technique to the other. To provide a direct diagnosis or a set of comprehensive features to untrained pathologists, several groups have developed machine learning tools,^20^ including deep learning, associated with either standard histopathology,^21^^–^^23^ or new optical technologies,^12^^,^^16^^,^^19^ including standard OCT^24^^–^^26^ to detect breast tumor margins. Another original approach is to use convolutional networks to transform endogenous optical images into H&E-like images by virtual labeling^27^^,^^28^ to facilitate the direct interpretation by the pathologist.

Recently, we developed and combined static and dynamic full field optical coherence tomography (FF-OCT)^29^^–^^31^ and demonstrated that these techniques can be of high interest for histopathological diagnosis. One advantage of this combination is that it captures non-destructively *en face* sections of tissues with a contrast resembling standard H&E histology, hence supposedly easily interpretable by histopathologists. Although similar, the obtained contrast is still sufficiently different and possibly richer than H&E histology. It still requires additional training and understanding to be well interpreted. We thus investigate here the potential of two machine learning algorithms to help and automate tumor diagnosis from static and dynamic FF-OCT images.

FF-OCT is a variant of OCT^32^ with superior lateral resolution and is able to acquire *en face* views of the sample in a single camera frame.^29^^,^^33^ FF-OCT captures light backscattered by a sample at a given depth^32^^,^^34^ and carries information on the three-dimensional (3D) tissue architecture (mostly extracellular matrix), which is significantly disorganized in cancerous biopsies.^35^^–^^37^ Endogenous light backscattering signal, even at relatively low spatial resolution, as measured in standard OCT, is highly predictive of the tumoral state of a sample,^38^ and tumor margins were successfully defined using OCT and machine learning.^24^^–^^26^ Recently, we developed dynamic FF-OCT (D-FF-OCT) that takes advantage of the intracellular dynamics of cells to add a new contrast depending on cell motility, metabolism,^30^ and can also reveal cell mitotic state.^39^ Combined with static FF-OCT, both the 3D structure and cell distribution and shape are recovered, offering a view of the sample resembling standard H&E histology.^31^ Static FF-OCT provides specific signatures of extracellular matrix, hence being similar to eosin staining, while D-FF-OCT allows visualization of nuclei (similarly to hematoxylin staining), cytoplasm, immune cells, and red blood cells. In contrast to histology, FF-OCT does not require tissue fixation or staining and allows measurement of a $1.3\times 1.3\;{\mathrm{mm}}^{2}$ region in a few seconds. It has the potential to offer a fast, 3D, label-free, wide field, non-destructive characterization of tumors, and detection of tumor margins directly in the operating room. A recent first study on 173 breast samples demonstrated that combining static and D-FF-OCT allowed rapid diagnosis of breast tumors during surgery with high accuracy around 90%.^40^

In this paper, we first performed a clinical pilot study in 35 patients (N=51 samples) with breast cancer to evaluate the clinical potential of combining static and dynamic FF-OCT in comparison to H&E histology. We demonstrated that FF-OCT offers enough similarities with standard histology to be straightforwardly translated to histopathology diagnosis. After a short training, two pathologists obtained a total accuracy of 88% (88% sensitivity and 87% specificity) by looking at static and dynamic FF-OCT. In contrast to the previous study,^40^ we demonstrated that combining static and dynamic FF-OCT allows imaging only a fraction (10% to 25%) of the specimen while obtaining similar good accuracy so rapid imaging below 10 min could be achieved in principle.

**Fig. 1:**
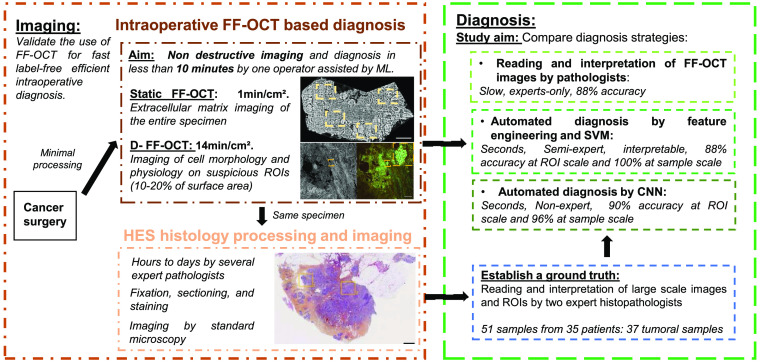
Workflow of this study and intraoperative FF-OCT based diagnosis. The aim of the study is to evaluate the use of static and dynamic FF-OCT for intraoperative breast cancer diagnosis and to compare manual and two automated diagnosis strategies using machine learning (ML). Imaging: Directly after excision and with minimal processing, a surgical specimen is imaged using static and dynamic FF-OCT, within several minutes. The same sample is then integrated to a common histology workflow for establishing a diagnosis by histopathologists. Diagnosis: We designed and compared three different diagnosis strategies using the FF-OCT dataset, with respect to the gold standard histology diagnosis. Direct interpretation of FF-OCT images by pathologists was performed, as well as automated diagnosis by FE and SVM or by CNN.

More importantly, we developed and compared two machine learning strategies to propose an automatic diagnosis of the breast tumors, for the first time with D-FF-OCT. The first approach, based on feature engineering (FE), aims to measure precise metrics such as collagen fiber disorder or cell density and classify samples based on the combination of these metrics. The second approach consists of automatic feature extraction using a custom convolutional neural network (CNN). Both approaches were applied on small regions of interest (ROIs) covering between 10% and 25% of each sample (representing above 1000 images in total) and obtained similar classification accuracy of 88% (89%, sensitivity, 86% specificity) and 90% (92% sensitivity, 85% specificity) at such scale, respectively. By aggregating the results of all ROIs to their respective samples (N=51 samples), we obtained an improved accuracy of 100% and 96% (94% sensitivity, 100% specificity). Both machine learning approaches thus demonstrated promising performance in automatic classification. A table summarizing all techniques and results obtained within this study can be found in Fig. 1 and in Supplementary Material.

Altogether, these results demonstrate that combining static and dynamic FF-OCT allows manual and automatic accurate cancer diagnosis, comparable with the H&E histology gold standard without tissue preparation and modification. We thus demonstrate the feasibility to obtain an automatic diagnosis on a surgical specimen directly in the operation room within 10 min. It has the potential to offer a non-destructive alternative to current intraoperative practices such as frozen section and touch preparation cytology.

## 2 Results

### 2.1 Static and Dynamic FF-OCT Allows Cancer Diagnosis by Medical Experts with 88% Accuracy

**Fig. 2:**
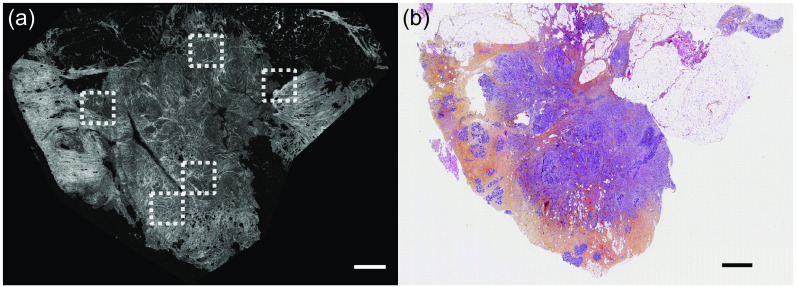
Comparison between static FF-OCT and histology images of the same entire specimen. (a) After specimen excision, a large field static FF-OCT image of the whole sample is acquired. For each sample, 5 to 20 ROIs (dashed white box) were manually selected and imaged with both static and dynamic FF-OCT (see Fig. 3). (b) Then, the sample was fixed, sliced, stained, and imaged according to H&E histology standards. Scale bars are 1 mm.

In this study, we imaged 51 samples, freshly excised from 35 fresh lumpectomy or mastectomy surgical specimens for intraoperative examination. For all specimens, one sample was taken in the tumorous area and, when possible, a second sample was taken far from the tumorous area to obtain healthy tissue. Immediately following the excision, the entire sample was directly inserted in the FF-OCT sample holder and imaged with the FFOCT microscope (CelTivity Biopsy System, Aquyre Biosciences). The static FF-OCT image of the entire sample (typically $2\times 1\;{\mathrm{cm}}^{2}$) was acquired [Fig. 2(a)] in <5  min. To save time and tissue freshness, instead of the whole sample, only 5 to 20 ROIs, i.e., 10 to 25% of the sample, were imaged with D-FFOCT. An ROI corresponded to one single field of view of the microscope (size $1.3\times 1.3\;{\mathrm{mm}}^{2}$) and the positions of the ROIs were manually selected to be representative of the different areas of the sample. One static and one dynamic FF-OCT image were acquired at each ROI (see Sec. 4). During this study, FF-OCT imaging time totaled 30 min to 1 h per sample, including acquisition and transfer of the raw data needed for research purposes. Nonetheless, in a real clinical context, without recording the raw data, similar procedure could be achieved directly inside the operation room in <10  min. Once the imaging had been performed, the samples were placed in histology cassettes, were fixed in formalin, included in paraffin, sliced and H&E stained (see Sec. 4). A diagnosis was performed by the pathologist by standard evaluation of all the H&E slides of the sample [Fig. 2(b)].

**Fig. 3:**
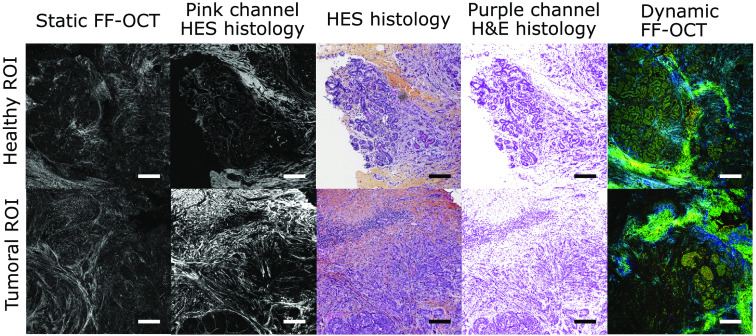
Comparison between static FF-OCT, dynamic FF-OCT, and histology images for a healthy and a tumoral ROI. The two ROIs (top and bottom rows) were imaged from the same sample diagnosed with IDC. Static (first column), D-FF-OCT (last column), and H&E (central column) images of a normal lobule within the sample are compared. To allow for easier comparison of FF-OCT and histology, we split the H&E images into their pink channel, displayed in gray to match FF-OCT color scale (second column) and into their purple channel (fourth column). Similar images (bottom row) in an IDC area are also compared. Scale bars are 0.2 mm.

Manual correlation and alignment of the large-field static FF-OCT image with the histology image allows to establish good correspondence between the two images, as shown in Fig. 2. By using the coordinates of the ROIs in the whole static FF-OCT image and by looking at the same coordinates in the histology image, we could obtain a side by side comparison of the static FF-OCT, the dynamic FF-OCT, and histology images for each ROI (Fig. 3). It became clear that the static FF-OCT image reveals the extracellular matrix (Fig. 3 left panels), hence being similar to eosin labeling (Fig. 3; second and third columns) in pink, while dynamic FF-OCT allows visualization of cells cytoplasm and nuclei (Fig. 3; right panels), hence giving a similar contrast to hematoxylin in purple (Fig. 3; third and fourth columns).

The two pathologists who performed the sample selection went through a blind analysis of the images deciding on the presence of cancer or not in the sample based on FF-OCT and/or D-FF-OCT images only. Pathologist P1 was already familiar with FF-OCT images since she participated in a study to evaluate FF-OCT performance on head and neck cancer diagnosis^41^ but used D-FF-OCT for the first time. The second pathologist P2 had no prior experience in any optical endogenous imaging technique. A short training was organized on four samples, where the two pathologists shared their interpretation of the FF-OCT and D-FF-OCT images and compared them with the corresponding H&E stained images. After these four samples, the two pathologists felt confident enough to blindly analyze the rest of the samples, due to the resemblance between FF-OCT and H&E histology. A set of features used by the pathologists to perform the diagnosis with the different modalities is described within the Supplementary Material.

The 47 remaining samples were presented to the pathologists and the reading was organized in two steps to compare diagnostic performance between static and dynamic imaging modalities (Table 1). First, only the high-resolution static FF-OCT image of the whole specimen [Fig. 2(a)] was used to propose the diagnosis, similarly to previous studies performed with static FF-OCT.^37^ An average accuracy of 85% (83% sensitivity, 91% specificity) was obtained (Table 1). We compared the diagnosis accuracy of simple static FF-OCT to a second strategy where we combined static and dynamic FF-OCT on a representative 10% to 25% fraction of each specimen. The localization of each ROI within the full sample was also given to the pathologists. Despite the partial imaging, a slightly higher accuracy of 88% (88% sensitivity, 87% specificity) was obtained (Table 1).

**Table 1:** Sensitivity, specificity, and accuracy of pathologists P1 and P2 in the differential diagnosis of 13 non-tumorous and 34 tumorous samples (47 samples in total). This table shows a comparison between a first reading where only a large field static FF-OCT image of the whole specimen is used and a second reading where static and dynamic FF-OCT were combined, but only on a few ROIs. 47 static FF-OCT scans of the entire specimens were used as well as 500 pairs of single ROI from these 47 samples for the partial static and dynamic FF-OCT diagnosis.

	Whole static FF-OCT	Partial Static and Dynamic FF-OCT		
	P1 (%)	P2 (%)	P1 (%)	P2 (%)
Sensitivity	77	89	86	91
Specificity	100	83	92	83
Accuracy	83	87	87	89

The addition of D-FF-OCT improved the performance of the two pathologists showing that D-FF-OCT highlights tissue features that are closer to the histology criteria the pathologists rely on to base their diagnosis. However, D-FF-OCT also caused misinterpretation from both P1 and P2 on two cases that were previously diagnosed correctly using FFOCT only, which demonstrates the interest of using both modalities and the drawback of partial imaging. In total, five wrong diagnosis were counted for each pathologist among which only three were common to P1 and P2 (discussed within the Supplementary Material). Therefore, improvement of the pathologists’ performance is expected with more training or with the help of automatic annotation tools. Nonetheless, isolated invasive cancer cells are more difficult to visualize with D-FF-OCT compared with histology, which was likely the cause of some errors of diagnosis and one particular target of automated algorithms.

Although these results are encouraging, they required training and time from expert histopathologists and could be improved by computer-based assistance. Efficient automatic diagnosis of FF-OCT images would refine, facilitate, and accelerate intraoperative interpretation, thus promoting clinical adoption.

Hence, in the rest of the article, we developed and tested two machine learning algorithms to perform automatic classification of the FF-OCT images and diagnosis of the samples.

### 2.2 Feature Engineering and SVM Analysis of Static and Dynamic FF-OCT Allow an Automatic Cancer Diagnosis and Identification of Decisive Features for Interpretation

**Fig. 4:**
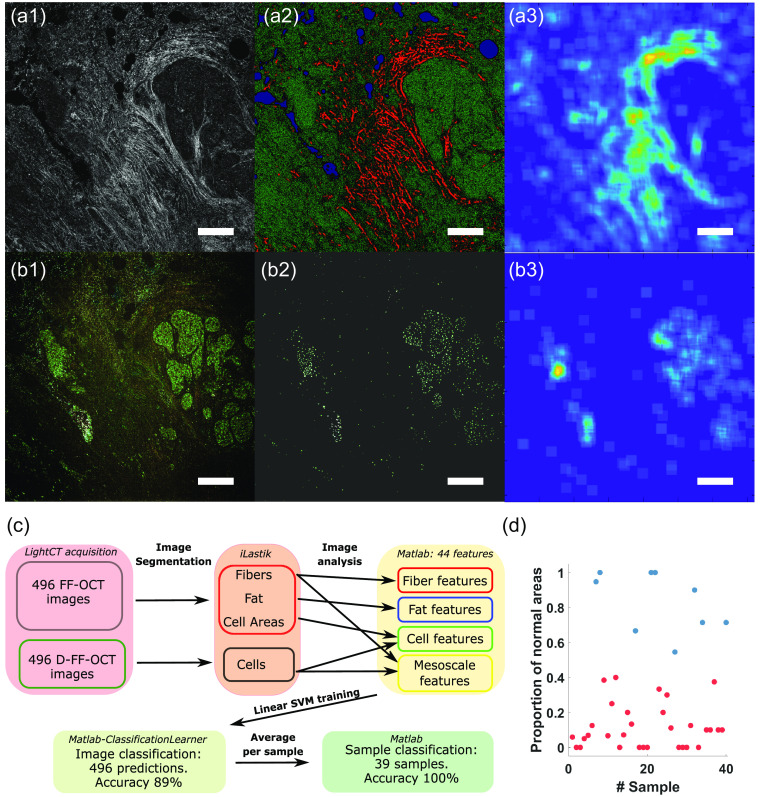
FE and SVM classification of breast cancer D-FF-OCT images. (a1) Static and (b1) dynamic FF-OCT images of a cancerous breast sample are analyzed using (a2), (b2) two random forest classifiers. (a2) FF-OCT image is segmented into fibers (red), cells (green), and fat/holes (blue). (b2) D-FF-OCT image is segmented into cells. (a3) Mesoscale fiber regions describe the region of high fiber density in the FF-OCT image. (b3) Mesoscale cell regions describe regions of high cell density in the D-FF-OCT image. (a2), (b2) The segmented images and (a3), (b3) the mesoscale images are used to calculate engineered features, such as cell and collagen fiber characteristics and size and shape of regions of high cell density. (c) Chart summarizing the processing of FF-OCT and D-FF-OCT images in order to classify each image and each sample using SVM. (d) Proportion of normal areas found for each healthy (blue) and cancerous sample (red) showing 100% separability between the two classes. Scale bars: 200  μm.

The first automated approach was to measure features of interest in the dataset via image analysis and use these features in a multidimensional machine learning model, linear support vector machine (SVM), to separate healthy from tumoral ROIs. We first selected (see Sec. 4) only the images where both static and dynamic FF-OCT modalities were available on the same ROI [Figs. 4(a1) and 4(b1)]. When the number of ROIs was too small (<5), the images from the corresponding sample were removed from the dataset. This resulted in a dataset composed of 496 static and 496 dynamic images from 39 samples (30 tumoral and 9 healthy). In each image, we aimed to measure features describing the cells and the extracellular matrix organization. We trained two random forest classifiers (one for static and one for dynamic FF-OCT) to segment individual cells and fibers [Figs. 4(a2) and 4(b2)] using the *ILastik* software^42^ (see Sec. 4). The segmented images were then analyzed using a second filtering step (see Sec. 4) to exclude some misclassified pixels. We also calculated mesoscale features, by segmenting regions of high-fiber, or high-cell, densities [Figs. 4(a3) and 4(b3)]. In total, combining static and dynamic FF-OCT images, we measured 44 features (listed in the Supplementary Material with associated references), including cell parameters (size and intensity), fiber parameters (density and organization), mesoscale organization, and fat content [Fig. 4(c)] that were previously characterized as potential cancer biomarkers (see Sec. 4).

For all 496 ROIs in the dataset, we therefore obtained 44 features that were used to classify each sample as compared to a ground truth defined as the histological diagnosis (see Sec. 4). We trained an SVM to classify each ROI [Fig. 4(c)]. We used a fivefold cross validation and penalized by a factor 3 the possibility of having false positives (healthy samples found as cancerous) to balance the dataset inhomogeneity (See Sec. 4 and Supplementary Material). The training set was therefore composed of all ROIs from 80% of the samples for each fold (see Sec. 4). With a linear SVM, we achieved a mean accuracy of 88±3% (sensitivity 89±4% and specificity 86±3%) and an area under the ROC curve (AUC) of 0.90±0.03 at the individual image level over the five models (see Supplementary Material). From the SVM model, a feature analysis allowed to measure the importance of each feature to separate the dataset and study the possibility of feature reduction. In total, we could reduce the number of features to 21 (see Supplementary Material for details and description of the kept features) without impact on the prediction accuracy of the network. The feature reduction had a marginal impact on the computation time and SVM performances but allowed for finding which parameters can better describe this specific dataset, hence helping to understand the measured changes in the tumoral samples. An important finding of the feature reduction is that the SVM approach needs features calculated from both static and dynamic images.

Finally, we used the predictions obtained at the ROI scale to classify the sample from which the ROIs were taken. Because of the imperfect prediction at the ROI scale (especially since the ground truth is not defined at the local scale here), we discarded the straightforward strategy of defining a sample as tumoral as soon as one ROI is tumoral, which would amplify the impact of false positives and would result in a low accuracy. Although this strategy will be further discussed, we tried to optimize the prediction strategy at the sample scale. Here, we computed the ratio of ROIs classified as normal versus all ROIs for each sample [Fig. 4(d)]. A clear separation between tumoral and healthy samples was observable. Hence, we applied a simple threshold on the ratio of normal ROIs fixed at 0.5. Healthy samples (blue dots) are all above this threshold, while tumorla samples are all found below. This resulted in a 100% accuracy segmentation between healthy and tumoral samples at the macroscopic scale. This result has to be mitigated (see Sec. 3) since the threshold above is necessarily arbitrary and quite specific to our dataset. Defining a general threshold would assume that during tumor excision a reasonably minimal and similar amount of healthy surrounding tissue is also removed.

### 2.3 Convolutional Neural Networks Combined with Dynamic FF-OCT Allow a Rapid Automatic Cancer Diagnosis and a Rapid Focusing on Potential Tumoral Regions

**Fig. 5:**
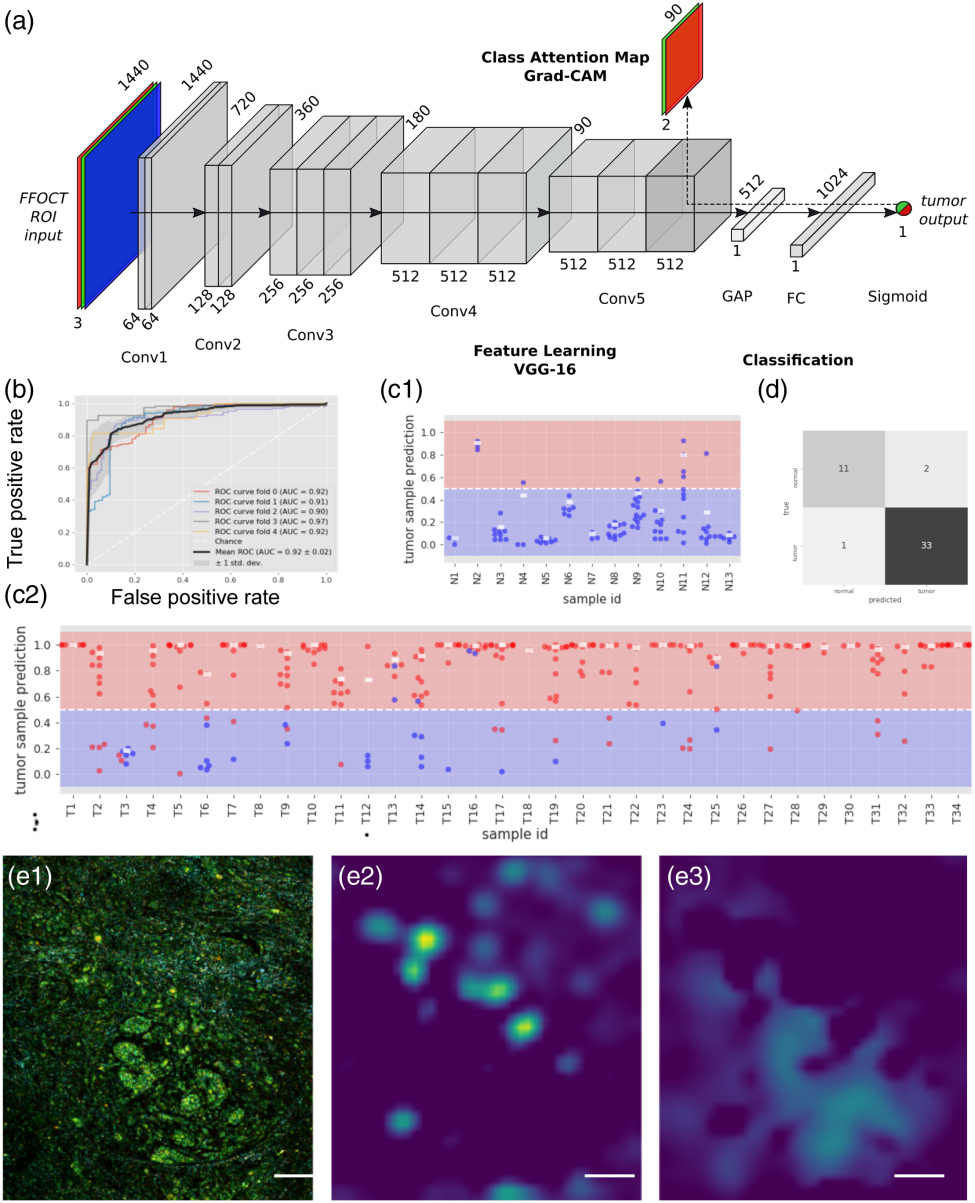
CNN classification of breast cancer. (a) Structure of the CNN used for D-FF-OCT CNN classification. The CNN can be used to provide a probability output at the end. An alternative is to propagate the gradients from the last convolutional layer using the GradCAM algorithm in order to retrieve some spatial information from the CNN. (b) ROC curves and AUCs of the fivefold cross validated models. (c) Tumor probability prediction by the CNN for each ROI in each sample, either (c1) healthy or (c2) tumoral. Each point represents an ROI where ground truth diagnosis is either healthy (blue) or tumoral (red). The white rectangle represents the aggregated tumor probability per sample, computed as the 90th percentile of the probabilities of the ROIs in a sample. (d) The sample scale diagnosis is found by applying a threshold at 50% for this 90th percentile. For each ROI, the Grad-CAM algorithm allows to recover attention maps. As an example, from an ROI showing healthy breast lobule surrounded by isolated infiltrating cancerous cells, correctly predicted as cancerous with 97% confidence by the CNN (e1), the tumor positive attention map (e2) focuses on the regions with the infiltrating cells, and the the tumor negative attention map (e3) shows healthy regions, including the healthy breast lobule. Scale bars are 200  μm in (e).

Our second approach was to explore a purely data-driven approach facilitated by the deep learning paradigm. We chose to fine-tune a pre-existing CNN, namely the VGG16 ^43^ architecture with weights pre-trained on the ImageNet dataset,^44^ to directly classify each ROI from the D-FF-OCT images. The rich variability present in the ImageNet dataset has been proven countless of times to provide a better initialization than more specific but less abudant datasets. For example, initialization with ImageNet was proven to be about 15% more efficient compared with other specific datasets in the case of breast cancer H&E histology.^45^ We modified the VGG16 to adapt its architecture to our problem [see Sec. 4 and Fig. 5(a)]. The network was trained on full resolution 1440×1440×3 RGB D-FF-OCT ROIs. In this section, we used in total 373 individual ROIs from 47 samples from which frame by frame annotation was performed by the histopathologist P2 (34 samples with a tumor, 13 without; see Sec. 4 for extended dataset description and correlation with histology). We used the ROIs from 80% of the samples (37 samples; 286 ROIs, 185 positive, and 101 negative) for training the network and used data augmentation (see Sec. 4) to obtain a sixfold increase of the training set size. The 20% remaining (10 samples) were used to test the CNN (see Sec. 4 for details) with a fivefold cross-validation to test the entire dataset ultimately. The CNN outputs a probability for the ROI to be in the tumoral part, and we defined a threshold at 50% above which the ROI is considered as positive. Using the fivefold cross-validation, we obtained a mean accuracy of 89±4% (sensitivity 88±4% and specificity 86±6%) and the area under the ROC curve (AUC) of 0.92±0.02 at the individual image level [Figs. 5(b) and 5(c)] over the five models.

Similarly to the FE strategy, we now want to interpret these results to perform a classification at the sample level taking advantage of the fact that the CNN outputs a tumor prediction probability (not only a class). Here also, there are multiple possible strategies that revolve around the assumption that normal samples contain only normal ROIs, and as few as one tumoral ROI makes the entire sample tumoral. Defining a sample as tumoral if only one ROI is found as tumoral (probability above 50%) would result in a total accuracy at the sample level of 85% (97% sensitivity and 53% specificity). By modifying the classification criteria at the ROI level and requiring a CNN output probability >80%, it is possible to increase the sample diagnosis performance to 89% (91% sensitivity and 85% specificity), but it also gives a large importance to false positives. Instead, in an attempt to balance out the misclassification errors present at the ROI level while also diverging as little as possible from the original assumption, we considered the tumor prediction probability for all ROIs. For each sample, we computed the 90th percentile of the probabilities of the presence of a tumor [white dots in Figs. 5(c1) and 5(c2)]. If the probability is above 50%, the sample is considered as cancerous. This results in a sample-wise accuracy of 94%, sensitivity of 97%, and specificity of 85%, translating to two false positives and one false negative [Fig. 5(C)]. Interestingly, the misclassified cases by the CNN were also wrongly diagnosed by the pathologists at the blind D-FF-OCT assessment.

Finally, because an accurate labeling at the microscopic scale is not always possible and meaningful, as well as to increase CNN interpretability, we developed another strategy based on the computation of activation maps of the CNN. Typically, in CNNs, convolutional layers naturally retain spatial information, whereas fully connected layers—usually present at the end of the neural network performing the classification task—do not, causing the loss of spatial information. However, this spatial information can be restored by locating the image area that triggered the CNN to decide which prediction to make [Fig. 5(a)]. In practice, we used the GradCAM technique^46^ (see Sec. 4). This results in a coarse localization of the class presence (here tumoral areas) in the initial ROI [Fig. 5(d1)], without the need for annotation at smaller scale. Positive and negative attention maps can be computed, showing either cancerous cells [Fig. 5(e2)] that invaded the stroma or healthy lobules [Fig. 5(e3)]. The activation map has a reduced spatial resolution of the same size as the last convolutional feature map of the network (leading to a pixel size of 45  μm). Besides veryfing that the model is not biased, building attention maps has also a clinical potential in drawing attention to specific parts of the image to assist the surgeon to classify the sample.

A summary of all diagnosis methods used in this study, their main features and accuracy, is presented in Table 2 to allow their rapid comparison.

**Table 2:** Summary of techniques and results obtained in the study. Only the accuracy is reported here, and the specificity and sensitivity are shown latter. N.A. means not applicable. The last two rows, and corresponding techniques, correspond to the main novelty of the article.

Imaging technique	Diagnosis method	Supervision level	Training set	Training time	Total time to diagnosis	Accuracy per ROI ($2\;{\mathrm{mm}}^{2}$)	Accuracy at sample scale ($2\;{\mathrm{cm}}^{2}$)
H&E	Histopathology diagnosis	Expert	Ground truth	Years	Hours to days	Gold standard (100%)	
Static FF-OCT	Whole static FF-OCT diagnosis by medical expert	Expert	Whole H&E image	Years + hours of new training for FF-OCT	2 min	N.A.	85%
Static and dynamic FF-OCT	FF-OCT diagnosis on 5 to 15 ROIs by medical expert	Expert	Whole H&E image	Years + hours of new training for FF-OCT	10 min	N.A.	88%
Static and dynamic FF-OCT	FE + SVM classifier	Semi-automatic	Whole H&E image	Minutes	15 min	88%	100%
Dynamic FF-OCT	CNN on medical expert annotation	Automatic	H&E ROIs	Hours	10 min	89%	94%

## 3 Discussion

To summarize, we demonstrated the potential and strength of combining static and dynamic FF-OCT to perform an accurate label-free and nondestructive histopathological diagnosis in breast samples, either by manual or automatic image interpretation. Interestingly, static FF-OCT offers a view of the extracellular matrix and tissue organization of the sample, and D-FF-OCT reveals cells within this matrix. The combination of both techniques reveals information comparable to H&E staining. In this work, we demonstrated the potential of combining static and dynamic FF-OCT to perform a fast, perioperative diagnosis to evaluate surgical specimens phenotype, and possibly to define tumor margins within a few minutes. In contrast to other state-of-the-art perioperative diagnosis methods, such as frozen sections and touch preparation cytology, FF-OCT is advantageously non-destructive, hence it can allow a second step of standard histology to confirm the results at a longer time-scale and has the potential to be more accurate. To further evaluate the potential of FF-OCT to be used as a perioperative tool, we additionally developed two methods of image processing and statistical learning and successfully brought more specificity and interpretability to these images. To our knowledge, this is the first time that machine learning algorithms are developed for D-FF-OCT. In the histopathology field, many machine learning algorithms have been developed on traditional staining, but applying them to FFOCT aims to facilitate the introduction of a high-potential imaging technique with a similar, but label-free and richer contrast mechanism in the field. In the end, although these results are preliminary and should be confirmed on bigger cohorts and different cancer types, we demonstrated that automatic diagnosis with FF-OCT compares well with manual diagnosis given by histopathological experts and should show increased performances as larger and more precise datasets will be generated.

Currently, one of the key limitations of FF-OCT based cancer diagnosis is that the D-FF-OCT image of large samples would require about half an hour of acquisition (see Fig. 1) while we aim to keep acquisition time below 10 min for intraoperative diagnosis. Within this time constraint, we had to image only a few ROIs per sample. This partial imaging procedure suffers from sampling bias if the ROIs are selected randomly. In this work, we demonstrated that this partial imaging was still sufficient to obtain diagnosis accuracy above 90%. We expect that by optimizing the imaging protocol (without recording the raw data mostly) and having the FF-OCT microscope inside the operation room would allow reducing the processing time down to 10 min, corresponding to the imaging time only. Besides, to reduce ROI selection bias, we could implement a two-step automated strategy. Since, static FF-OCT imaging is faster (1 min for $1\;{\mathrm{cm}}^{2}$ of sample), it could be used as a first row of interpretation and drive the selection of the ROIs where to apply D-FF-OCT for further investigation. For future studies, we aim to develop a first CNN trained on static FF-OCT image of the whole sample^47^ to automatically define a few ROIs where the D-FF-OCT will be acquired to increase the accuracy of the diagnosis. We can also expect that progress in camera technologies and in real time processing of the D-FF-OCT image via GPU computing will help reducing the imaging time and will soon enable one to record the D-FF-OCT of the entire sample in a few minutes.

In this study, we developed and tested two machine learning algorithms in which strategies and outputs are complementary. If both techniques are hard to compare quantitatively, they are likely to target different applications.

The FE approach is based on the computation of known meaningful metrics such as collagen fiber characteristics or cell morphological parameters that could be used directly on top of the classification. Hence, it is likely to be more appropriate for exploratory diagnosis, where the quantification of these metrics can discriminate between different diagnosis (e.g., different tumor types). Because the FE approach relies on averaging cell and fiber parameters, this approach would converge more easily if the tumor is homogeneous with such parameters centered around well defined values. In this study, we realized that the computation of these metrics is still rather imprecise (e.g., it is extremely complicated to make sure that 100% of the cells are segmented and that their diameter is correctly sampled at the optical resolution). Besides, some parameters (e.g., extracellular matrix disorganization) are not necessarily well defined at the microscopic scale. So that the FE approach did not perform well when the spatial scale is reduced. This is expected given that the FE approach is quite similar to the reading method of the histopathologist, who most often requires reviewing large portions of the sample to provide an accurate diagnosis. In general, improving the metric computation, defining new useful metrics, and increasing the spatial resolution might help to increase the sensitivity of this approach. Among others, the FE approach could be greatly improved if the aspect of cell nuclei and the number of mitotic cells, key metrics in standard histopathology,^48^^,^^49^ could be automatically quantified. In previous work, we demonstrated that D-FF-OCT performed at high resolution is able to measure the aspect and dynamics of nuclei^31^^,^^50^ and measure the mitotic state,^39^ suggesting that this measurement could be automated. Nonetheless, increasing the resolution decreases the field of view and thus increases the imaging time if a full sample is acquired. The best compromise between resolution (and more precise computation of cell metrics) and imaging time should be defined depending on the application.

Our second strategy was based on CNNs. The CNN approach works without *a priori* knowledge of the tumor biology, and the same network structure can be applied to any other D-FF-OCT dataset as long as sufficient annotation is available. The CNN would reach better accuracy with a greater dataset size and label homogeneity. It would, therefore, be more recommended for large and well annotated datasets or when drawing the tumor margins with high resolution is required. Nonetheless, the CNN approach is less interpretable, and because it does not rely on any general feature of tumors, it is likely that a new network should be retrained from scratch for any new tumor type, whereas the FE approach could potentially identify general biomarkers. We also believe that the Grad-cam algorithm that defines attention maps, i.e., subregions that were the most meaningful for the network to choose a diagnosis, could be an important help to rapidly draw the attention of surgeons or histopathologists on suspicious ROI. Because the CNN approach is unbiased as compared with human interpretation, the use of attention maps could also improve our knowledge of tumorous processes by pointing out small details as potential tumor markers, proper to our imaging modality.

It has to be kept in mind that the two automated methods produce a probabilistic prediction (which we here mostly thresholded at 50% to perform a prediction). Therefore, it is easy to associate their predictions with a level of confidence hence giving the possibility of deciding which samples can be analyzed automatically and which sample should concentrate the attention of expert histopathologists.

It is interesting to see that many label-free imaging techniques have been associated with machine learning to provide automated cancer diagnosis with excellent and comparable accuracy. If this is complicated to provide a quantitative accuracy comparison between these techniques since the samples are different, it is still interesting to compute here differences in the raw signals used for the prediction.

Fluorescence lifetime imaging detects molecular changes in the environment of endogenous fluorophores so fluorescence decay is slower in tumoral regions and faster in adipocytes. It is, therefore, partly molecular-specific, it is a surface and rather low resolution method and has shown automated breast cancer diagnosis with accuracy of 97% in breast lumpectomy specimens^51^ and around 90% in an intraoperative setting using a handheld probe.^16^ OCT has the particularity to provide an axial and potentially volumetric view of the specimen, hence being able to capture tumors in depth, whereas the other techniques, including histology mostly focuses on sample layers close to the surface. OCT (e.g., accuracy of 81% in some skin cancer as early as in 2007,^52^ 93% in breast sample with volumetric OCT^53^) only relies on light scattering changes associated with denser, or more heterogeneous extracellular matrix, and can be made sensitive to polarization so to measure the collagen fiber order (e.g., accuracy of 93.5%^54^ in breast or 95% in skin.^55^). It is a rather low resolution method but is fast and can be compacted in handheld probe devices in intraoperative settings (accuracy of 97% to detect canine soft tissue sarcoma^56^). Static FF-OCT (accuracy of 95% in basal cell carinoma^47^) and polarization sensitive FF-OCT (around 88% accuracy in breast cancer^57^) are also similar solutions with higher resolution but transverse detection.

Multiphoton endogenous microscopy (e.g., 97% accuracy in differentiating stage I invasive ductal carcinoma (IDC) from normal human breast^19^) is also very promising as it provides high resolution images with molecular sensitivity so a map of the tumor can be drawn. It requires high intensity lasers and it is slower compared with OCT and FLIM, so it might be more appropriate for high resolution precise characterization of tumor samples rather than for large scale inspection of samples. In contrast, combining static and dynamic FF-OCT as it is done here can combine scattering and organization of the extracellular matrix with the morphological and metabolic parameters of the cells. One advantage of using automated detection by the combination of static and dynamic FF-OCT is that it gives a good compromise between the imaging speed and large scale capability while keeping cell resolution and some molecular information in the raw signal detection.

It is interesting to notice that all the imaging techniques cited above present excellent accuracy scores that should make them all more efficient quantitatively than intraoperative histology techniques, and it is hard for the authors to understand why none of them has yet emerged as a new gold standard. We believe that these imaging techniques as well as machine learning are now mature enough to be fully adopted and trusted by the medical community, even though specific studies should yet to be done for all tumor types. Yet, we acknowledge that an important ethics issue that should be addressed collectively is how to address the responsibility if a wrong diagnosis is made by automated algorithms. We believe this is therefore important to associate a diagnosis to a given confidence score and to maintain a second-hand validation by histopathologists everytime the confidence reaches a given threshold. Another interesting potential solution to export such new optical imaging techniques is the solution developed by the Ozcan’s group on autofluorescence imaging,^27^ where a CNN was trained not to perform a diagnosis but to transform an original autofluorescence image into a virtually H&E-labeled image that could be directly interpreted by pathologists.^27^ Due to the similarity between H&E labeling and the combination of static and dynamic FF-OCT, we believe that such transformation should be feasible and it will be the subject of future investigations.

Overall, we demonstrated that the computer-aided combination of static and D-FF-OCT reaches the standards of breast histopathology while being nondestructive, label-free, and free from freezing and slicing artifacts. We forecast two main contributions in histopathology. During intraoperative diagnosis where CNN could help deciding whether the entire tumor was removed in only a few minutes, without the direct need for a pathologist during the operation, with an accuracy not optimal but higher than standard intraoperative techniques such as frozen sections. Finally, the sample preservation enables to keep the sample for later more precise validation diagnosis and characterization by hsitopathologists. For such application, the CNN approach seems the most promissing as it could help focus the attention on suspicious regions of the sample and could perform a diagnosis without *a priori* knowledge about tumor biology. A second contribution could be to reduce the workload of pathologists by assisting diagnosis on samples with high confidence score while leaving others for manual traditional inspection. In this case, the FE method could be of interest as it provides a set of interpretable features that can be directly used to refine or validate the diagnosis.

More importantly, we believe the nondestructive aspect of FF-OCT, and other label-free techniques, is fundamental to go even beyond standard histology, because it allows combination with other techniques, including molecularly specific optical techniques (such as fluorescence or Raman spectroscopy) or destructive tumor genotyping. Multimodal imaging could thus identify both the spatial organization of a tumor and its molecular or genetic characteristics. As the cells are still alive during FF-OCT imaging, and as D-FF-OCT can monitor cell metabolism, an interesting individualized follow-up approach would be to perform chemotherapy treatment on the extracted specimen and evaluate the response from the metabolic changes of the cells within. Depending on the response of the specimen, it could orient the use of one molecule instead of another before starting to treat the patient. As histopathology is being moved by digital transformation, computer-aided FF-OCT must quickly develop and confirm its potential on larger datasets and various tumor types to be part of the transformation.

## 4 Methods

### 4.1 Sample Collection

In total, 35 patients admitted at Gustave Roussy Breast Surgery Department between June and August, 2017, for a mastectomy or lumpectomy were enrolled in this study, from which 51 samples were collected. All surgeries were performed at the Institut Gustave Roussy (Villejuif, France). The Institutional Review Board approved the study, and informed consent was obtained from all patients. Once the surgical specimen arrived at the Pathology Department, when confirmed that tissue extraction would not impact standard processing of the specimen, none, one or several specimens were taken in the tumorous area and none, one or several specimens were taken far from the tumorous area to sample healthy tissue. The heterogeneous tissue sampling resulted in 37 tumoral samples and 14 healthy samples according to the histology diagnosis. The most represented cancer type was IDC, but three other types were reported (see Table 3).

**Table 3:** Study set composition according to histology-based diagnosis.

Main diagnosis	Number of samples
Healthy	14
IDC	28
Invasive lobular carcinoma	5
DCIS	3
Lobular carcinoma *in situ*	1

### 4.2 Sample Imaging

For all samples, the static FF-OCT image of the entire specimen (typically $2\times 1\;{\mathrm{cm}}^{2}$) was acquired first. The Z-plane was chosen at 15  μm below the glass coverslip surface to maximize FF-OCT signal and avoid coverglass signal fringes artifacts. Unfortunately, imaging the full sample volume would require too much time at this stage.

Then, on average 11 ROIs corresponding to a single camera field of view of $1.3\times 1.3\;{\mathrm{mm}}^{2}$ were manually selected by the person in charge of imaging to be representative of the different areas of the sample. Unless an error or oversight was made by the imaging person, one static FF-OCT image and one dynamic FF-OCT (D-FF-OCT) image were acquired at each ROI and the raw data of the D-FF-OCT images was saved to allow custom processing, as used in the FE method (see dedicated Sec. 4 section). In total, 540 pairs of static and dynamic FF-OCT were acquired from the 51 samples, among which 500 were used for the blind analysis at the sample scale and 373 were later annotated by pathologist P2 at the ROI scale.

### 4.3 Ground Truth Definition and Definition of Reduced Datasets for FE and CNN Approaches

The performance of the pathologists’ interpretation based on the FF-OCT images was measured as referred to the standard H&E histology-based diagnosis of the same sample. As explained hereafter, only subsets of the original dataset could be used for each automated analysis:

• For the FE analysis, we included samples under three criteria. A sample was excluded if less than five ROIs had been acquired with complete data. Furthermore, for each ROI, one static and its corresponding dynamic FF-OCT image should be available (without translation), as well as the raw D-FF-OCT data. It resulted in 496 static and 496 dynamic FF-OCT images from 40 samples (31 tumoral and 9 healthy). In the FE analysis, the sample histological diagnosis is passed on to all the ROIs to define the ground truth for each ROI, resulting in a ground truth with some errors. We emphasize that the first level of prediction is made at the ROI level so the dataset size here is 496 raw images.
• In contrast, the CNN approach only relies on the dynamic FF-OCT images. Nonetheless, to obtain convincing results with this approach, histopathologist expert P2 was asked to label all the individual dynamic FF-OCT images based on his interpretation and correlation with the H&E histological slide. We converted the expert P2 annotation into a binary label indicating the presence of a tumoral part in each ROI. However, not all images could be labeled with high confidence, so the dataset was reduced to 373 dynamic FF-OCT images from 47 samples (34 with a tumor and 13 without). Here, we used data augmentation by replicating some raw images while changing the contrast and/or by applying geometrical transform (horizontal or vertical symmetries)—see dedicated section in Sec. 4. Hence, the final datasetsize corresponds to 2238 images.

### 4.4 Comment on Class Imbalance

Although we regret the class imbalance in our study with about three times more positive cases than healthy cases, we want to stress that it is not possible to invovle healthy patients for such invasive surgeries. The healthy cases correspond to regions far from the tumoral region when complete breast ablation was necessary for medical reason. Nonetheless, healthy samples should be more homogeneous than the tumoral ones, and we already accounted for this class imbalance in our study, so we hope we have minimized the effects of class imbalance.

### 4.5 FF-OCT Setup

A commercial Light-CT scanner, manufactured by CelTivity Biopsy System (Aquyre Biosciences), was used. It is a Linnik interferometer equipped with microsope ojectives (Olympus, UMPLFLN 10XW) immersed in silicone oil. It uses a broadband LED source (104 nm full width at half maximum, 565 nm central wavelength), thus providing a 3D resolution of 1.2, 1.2, and 1.0  μm for the X, Y, and Z axes, respectively. X, Y, and Z motorization allows axial and transverse scanning. The FFOCT signal was recorded using a high speed, high full well capacity (FWC) camera (Quartz 2A750, Adimec, 2 Me of FWC, 700 frames per second maximum). Image acquisition was performed at 150 frames per second to reduce the light power and avoid thermal damage to the samples. Static FF-OCT acquisition was performed using four-phase modulation induced by a piezoelectric actuator controlling small translations of the reference mirror. As a consequence, an FF-OCT single field of view required the acquisition of at least four images and typically 20 images with five times accumulation. Including scanning time, the FF-OCT imaging speed of the Light-CT scanner is $1\;\min.{\mathrm{cm}}^{-2}$. For D-FF-OCT acquisition, the mirror position is not modulated, and the system records the fluctuations arising from the motion of scatterers inside the coherence volume over a few seconds. Typically, 1000 images are acquired in 6.7 s. Then, the time series of each pixel is processed by FFT and results in an RGB value assignment per pixel, where the R-value is the FFT summed contribution over the high frequency range (5.4 to 25 Hz), the G-value is the FFT summed contribution over the medium frequency range (0.6 to 5.4 Hz), and the B-value is the (0 to 0.6 Hz). These frequency bands were selected and fixed during the first implementation of D-FF-OCT within the Light-CT scanner so it divides the FFT spectra of typical cell signals in three parts of equal energy and so the RGB images are well contrasted.^58^ Including scanning time, the D-FF-OCT imaging speed of the Light-CT scanner is $14\;\min.{\mathrm{cm}}^{-2}$. Saving raw data further lengthen the D-FF-OCT imaging duration. During the study, we kept the imaging duration below 30 min to reduce the elapsed time between resection and imaging so the tissue freshness was preserved. Hence, a good quality D-FF-OCT signal was guaranteed and the typical histology processing is as minimally altered as possible. This is why we performed ROI sampling instead of acquiring the whole D-FF-OCT image.

### 4.6 Signal Processing to Construct Dynamic FF-OCT Images for Feature Engineering

Before using the FE approach, we recalculated dynamic FF-OCT images using the raw data using a more recent processing pipeline,^39^ not yet implemented in the LightCT-scanner. We calculated color images in the HSV (hue–saturation–value) color space in which, contrary to the red–green–blue (RGB) color space, it is possible to assign a physical property to each of the three channels for quantitative visual interpretation. The idea is then to attribute a color for each pixel depending on the characteristic time period or frequency of the dynamic signal. Each individual pixel can be thought of as a sum of subcellular random walks with a typical covariance function depending on the motion type (e.g., diffusive and hyperdiffusive).

We started by computing the power spectrum density (PSD) using Welch’s method for each pixel and then used an L1 normalization on each PSD. Then, the hue channel was computed as the mean frequency (which is simply the dot product between the normalized PSD and the frequency array). The values were then inverted and rescaled between 0 and 0.66 to go from blue (low frequencies) to red (high frequencies).

Saturation was computed as the inverse of the normalized PSD bandwidth. As a consequence, the saturation channel carries the frequency bandwidth information. The saturation map is then inverted and rescaled between 0 and 0.8. The broader the spectrum, the lower the saturation. White noise has a broader bandwidth and will therefore appear greyish instead of colored.

Finally, the value is calculated using the cumulative sum on small time windows to improve the signal-to-noise ratio:^59^

$${\overset{¯}{I}}_{\mathrm{dyn}}(r)=\frac{1}{N}\underset{i}{\sum }\max(|\mathrm{CumSum}(I(r,t_{\left[i,i+\tau \right]})-\overset{¯}{I}(r,t_{\left[i,i+\tau \right]}))|),$$ (1)

where CumSum is the cumulative sum operator, N is the total number of sub-windows, τ is the sub-windows length so $t_{\left[i,i+\tau \right]}$ is the time corresponding to one sub-window and $\overset{¯}{I}(r,t_{\left[i,i+\tau \right]})$ is the signal mean on the sub-window. Images were computed with τ=16.

The resulting HSV image is finally converted into an RGB image for display purposes.

### 4.7 H&E Histology Preparation and Imaging

All imaged samples were fixed in 10% buffered formalin, embedded in paraffin, sectioned at 3  μm, and stained with hematoxylin, eosin, and saffron (HES).

Histological sections were scanned at 20× magnification with a NanoZoomer C9600 scanner (Hamamatsu Photonics, Massy, France) for interpretation and archiving. For the correlation, histopathologist P2 together with an FFOCT technology expert from LLTech, decided on the angle to apply to the histology image to obtain the same orientation as the FFOCT large-field image. Then, well-oriented histology image was displayed on one screen while the ROIs were consecutively displayed on a second screen. Both histology and FFOCT images were zoomed in and out to establish the correlation.

### 4.8 Tumor Detection Using Feature Engineering and SVM

#### 4.8.1 Segmentation of cells and fibers using iLastik

The first step of the engineered features analysis on breast samples was to perform cell and fiber segmentation in the images, using *iLastik*, a free segmentation software.^42^ Extensive description of parameters used in Ilastik as well as illustration of the segmentation workflow is proposed in the Supplementary Material. Briefly, *iLastik* is a relatively intuitive machine-learning tool based on random forest classifiers. For each channel of D-FF-OCT images, and for FF-OCT images, pixel neighborhood is characterized by a set of generic nonlinear spatial transformations calculated by the software and is compared with a training set of pixels, manually drawn on images from the dataset. For both FF-OCT and D-FF-OCT datasets, the training step was performed on eight images (five from cancerous samples and three from healthy samples). For D-FF-OCT, we defined three classes: cells, between cells, not cells (fibers, noise, fat, etc…), out of which only the cell class was used for cell segmentation. Fpr FF-OCT, we used four classes: fibers, around fibers, cells (and noise), fat (and holes/slicing artifacts). The around fibers class was used to segment individual fibers and not only the regions of high fiber density. The cells class contains most of what is not fibers, including extracellular matrix and cells. The fat class segments dark pixels in circular regions, as observed in fat regions of the breast [Figs. 4(a1) and 4(a2) in blue]. Unfortunately, it also finds a few holes in the sample, caused by slicing, folding artifacts, or bubbles.

In total after these learning steps, of about 30 min, each FF-OCT and D-FF-OCT image can be segmented in about 10 s.

#### 4.8.2 Further image analysis and feature calculation

The two sets of segmented images were then opened and analyzed using image processing (Matlab, MathWorks). From the D-FF-OCT images, we target individual cells, and some of their morphological parameters that we expect to be modified in a tumor, such as cell density,^60^ cell size,^61^ or shape.^62^ We also measured their light scattering properties^63^ and their motility.^62^ From iLastik segmented images, we detected and characterized all eight-connected objects using the regionprops function (Matlab, Image Processing Toolbox), which we estimate to correspond to single cells. We obtained hundreds of groups of pixels, we will refer to as cells in the rest of the section, although there can be a few artifacts within these objects. We additionally filtered all cells with an area below 20 pixels ($20\;\mu m^{2}$, corresponding to a cell radius below 2.5  μm) to filter out some classification errors. For each cell, we extracted its diameter, eccentricity, mean intensity on each RGB channel, and the spatial heterogeneity of intensity (standard deviation of pixels within the cell). We also computed the total cell density. For each image, we could obtain one histogram of the values of each parameter (e.g., of cell diameter). In order to use a reasonable number of parameters in the SVM, we only used the average and standard deviation of the obtained histograms.

In addition, we measured what we called mesoscale cell features to have a measurement of the spatial organization of cells at an intermediate level. For example, in healthy breast samples, relatively high cell density can be found in acinii, but cells are highly organized in a rather circular region with a central lumen. In ductal carcinoma *in situ* (DCIS), the acinii shows a modified shape with a progressive invasion of the central lumen.^64^ It is therefore important to quantify whether the high cell density regions contain small, well organized cells (as in healthy samples) or extended cells with an abnormal shape. To do this, we used the D-FF-OCT segmented image and convolved it with a 60 pixel wide square, which thus gives an image of local cell density (Fig. 4). From this map, we segmented all pixels with a local density above 0.05 (arbitrary) and detected all eight-connected objects. We then extracted the number of high density areas, their surface, and eccentricity. We finally counted the number of all cells whose positions are outside these regions of high cell density, aiming to track for tumor cells migrating outside the ducts.

From the FF-OCT images, we mostly target the stromal collagen fibers since their organization^65^ and light scattering properties^38^ are expected to change in cancerous samples. We also measured some properties linked with cell regions and fat regions to add information to segment the images. We first used the segmented images corresponding to fibers. We detected and characterized all eight-connected objects, corresponding to single fibers, and filtered out all regions of area below 100 pixels. We measured each fiber area, eccentricity, orientation, and intensity, as well as the fiber density and extracted the mean and standard deviation of each parameter in an image. We then calculate mesoscale fiber features by convolving the segmented fiber image with a 60 pixel wide square and by segmenting all pixels with local fiber density above 0.1. We kept the area, eccentricity, intensity, and number of these regions of high fiber density. We then used the image segmented by cell region to calculate the intensity histogram in these regions. We finally used the segmented image of fat to calculate the proportion of fat in the image. All features are summarized in a table in the Supplementary Material.

#### 4.8.3 SVM training

From the previous section, we obtained a matrix with 44 features for all 496 images in our dataset. The idea behind the SVM classification is to find an (N−1) dimensional hyperplane that best separates the dataset between healthy and cancerous images. If none of these features alone can clearly separate the dataset, the SVM will find a linear combination of all these parameters that best separate the dataset.

When a feature was missing (e.g., if no region of high cell density was found in an ROI), the value 0 was attributed.

For training the SVM, we attributed the histological diagnosis to all images of each sample. We could test several models (e.g., non-linear models) to perform the image classification (Matlab Classification Learner toolbox, MathWorks) but decided to keep a simple linear SVM to reduce the risk of overfitting (given the small dataset) and to keep the model interpretable. As mentioned in the main text, the dataset labeling is highly asymmetric; first, we have about three times more cancerous images than healthy images. Second, images from healthy samples should all be healthy, but images from cancerous samples can be either healthy or cancerous. In order to reduce overfitting (e.g., always predicting cancer should give 75% accuracy), we made the SVM learning asymmetric by penalizing the false positives by a factor 3.

#### 4.8.4 Feature reduction

To learn which features are the most relevant for the segmentation of tumoral versus healthy ROIs, we apply a neighborhood component feature selection algorithm. It consists of learning a feature weighting vector from neighborhood component analysis (NCA)^66^ by maximizing the expected classification accuracy with a tunable regularization parameter term.^67^ This technique is more robust to the presence of irrelevant features and to dataset normalization and hence is frequently used for feature selection prior to SVM classification. A first step to learn the optimal regularization parameter by gradient ascent technique is used, and the NCA model is fitted to all data using this optimal parameter in order to obtain the feature weights. The obtained feature weights were ranging between 0 and 2, and we selected only the features with weight above 0.05. It results in selecting 21 features underlined in green in the corresponding table in the Supplementary Material. Then, a similar SVM classification uses only these 21 features instead of the initial 44, but we found only marginal differences in terms of speed and accuracy of the SVM.

### 4.9 Tumor Detection Using Fine-Tuned CNN

#### 4.9.1 Description of the CNN architecture

The CNN architecture is similar to the VGG16^43^ architecture with weights pre-trained on the ImageNet dataset^44^ but with small modifications. We removed the classifier part and added a global average pooling (GAP) layer followed by a fully connected layer of 1024 neurons and an output neuron with sigmoid activation. The GAP layer allows network inputs of different sizes since it reduces each activation map to a single value, it results in a 512-dimensional vector bottleneck between the feature extracting convolutional layers and the dense classifier layers. Another advantage of GAP is the reduction of network parameters, which improves generalization and is particularly useful in the case of small scale data sets. Following the same reasoning, we chose only one fully connected layer. With the presented configuration, we obtain a network with ∼15 million parameters of which 500 thousand correspond to the classifier and the rest to the pre-trained weights.

#### 4.9.2 Training

The CNN was trained for a day on one TITAN Xp GPU card having 12 GB of graphic memory, 7 min per epoch, with early stopping, best model recorded after epoch 104 (so 12 h), some IO overhead is introduced by the generation of batches on the fly used keras with tensorflow 1.5 backend.

The network was fine-tuned by minimizing the binary cross-entropy loss using the stochastic gradient descent optimizer with a learning rate of 1e−4 and momentum of 0.8 on mini-batches of size 3 (due to memory constraints).

Since we opted for fine-tuning, training time is significantly reduced compared to a training from scratch approach: consequently the phenomenon of overfitting occurs much faster. To avoid training beyond the optimal model, we have set two stopping conditions: (i) validation loss has not improved in the last 100 epochs or (ii) training accuracy has already reached 100%. With this, training lasts around 200 epochs and the optimal model is found somewhere between epoch 80 and 150 (depending on the data split and initialization, so depending on the random state). Training time is around 6.5 min per epoch and 20 h per experiment. Thus, conducting a fivefold cross-validation experiment took around two and a half days (64 h); note that in these delays we also included the lag introduced by logging performance metrics, as well as the overhead introduced by reading the image batches from the disk, and not only training (i.e., forward and backward propagation). Experiment tracking was made possible with the software Neptune (Neptune.ai), which helped organize and compare the performance of over 200 experiments conducted for this project, therefore, allowing us to choose the optimal hyperparameters in an exploratory fashion.

#### 4.9.3 Data

The network was trained on full resolution 1440×1440×3 RGB fields of view with binary labels indicating the presence or absence of tumorous tissue, obtained from the pathologist’s refined annotation per ROI. An important detail is that in tumorous ROIs, there might be portions of the image resembling healthy tissue.

The dataset partitioning into training and test sets was performed in a stratified manner with respect to the global per-sample diagnosis; in terms of size, 80% of the samples ($n_{\text{train}}=37$, $n_{\text{train-tumor}}=27$, $n_{\text{train-normal}}=10$) used for training and the remaining 20% ($n_{\text{test}}=10$, $n_{\text{test-tumor}}=7$, $n_{\text{test-normal}}=3$) for evaluating the performance. However, this stratification strategy does not ensure the exact same distribution of classes at ROI level, as each sample has a different number of acquired ROIs; this results in 286 fields of view for training (185 positive and 101 negative) and 87 for testing (60 positive and 27 negative). To compensate for the class imbalance, an importance penalization of the loss function was applied for each ROI, which is also known in the literature as class weight. In our case, the healthy ROIs are less numerous so they will have a higher weight (i.e., 1.5 for healthy ROIs and 0.75 for cancerous ROIs).

In terms of data augmentation, i.e., artificially increasing the number of data points by applying relevant transformations to the existing data, we applied contrast stretching, with three look up tables per FOV, together with vertical and horizontal flips, which expanded the training set by up to six times.

#### 4.9.4 Quantitative performance

With a probability threshold set at 50% for ROI diagnosis, we obtained a per-FOV accuracy of 90% which corresponds to 92% sensitivity and 85% specificity. Another metric that is worth mentioning, due to its lack of dependence on the probability threshold, is the area under the ROC curve (AUC), which is equal to 0.94. For per-sample metrics, out of 10 samples in the test set, 7 of which were cancerous and 3 healthy, there was only one misclassification, which is a normal sample predicted as tumorous; this translates to 90% accuracy, 100% sensitivity, and 67% specificity

To aggregate the per-ROI predictions to a global per-sample diagnosis, assigning the maximum tumor probability prediction to the sample would be the most straightforward approach. This would translate to “if a sample contains at least one ROI with a tumor, then the sample is cancerous.” This approach is however overly sensitive to outliers. On the other hand, the average and median are not suitable either because a bimodal distribution is expected (i.e., a sample most likely contains both healthy and tumorous FOVs), in other words small tumorous areas would be missed or cancel out good prediction. Therefore, we chose the 90th percentile as a good trade-off between the mean and the maximum aggregations. This would translate into the probability value that 90% of the ROIs fall into.

In order to validate the method and ensure model correctness, we ran a fivefold cross-validation experiment with the same hyper-parameters and trained five models on partitions of 4/5 of the samples and tested their performance on 1/5 of the samples, respectively, hence keeping the same 80/20 train/test ratio at each run.

#### 4.9.5 GradCAM algorithm and attention maps

The receptive field represents the zone (or patch) of the input image that a CNN feature is computed upon. In classical convolutional arhictectures, like VGG16 used in the present work, that are composed of convolutional and pooling layers, the size of the receptive field is directly proportional to its corresponding layer depth, allowing to learn progressively more complex features. Receptive fields of pooling layers: 6×6, 16×16, 44×44, 100×100, and 212×212. The GradCAM algorithm uses the gradient information flowing into the last convolutional layer of the CNN [Fig. 5(a)] to assign importance values to each neuron for a particular class. The averaged gradients flowing back from a chosen class output neuron to a previous layer (usually last convolutional layer) act as weighting factors for each activation map; the final result being a linear combination between the weights and filter activation maps.

Attention maps: sum of last convolutional layers of the last two convolutional blocks in VGG, i.e., the third layer of the fifth (last) and fourth blocks. We chose to add the attention map of the second-to-last block to offer a finer detail of the segmentation due to its increased resolution, i.e., 180×180 as compared with 90×90.
