# Supplementary material for: Automatic diagnosis and classification of breast surgical samples with dynamic full-field OCT and machine learning
Source: J Med Imaging (Bellingham). 2023 Jun 1;10(3):034504. doi: 10.1117/1.JMI.10.3.034504 (PMC10234284; doi:10.1117/1.JMI.10.3.034504)
Supplement: Supplementary file 1 [file JMI_010_034504_SD001.pdf]

Supplementary Materials: Automatic diagnosis and classification of breast surgical samples with dynamic Full-Field OCT and machine learning.

Jules Scholler[1,*], Diana Mandache[2,3,*], Marie Christine Mathieu[4], Aïcha Ben Lakhdar[5], Marie Darche[6], Tual Monfort[1], Claude Boccara[1], Jean-Christophe Olivo-Marin[3], Kate Grieve[6,7], Vannary Meas-Yedid[3], Emilie Benoit a la Guillaume[2], and Olivier Thouvenin[1]

Basic optical configuration behind the LightCT microscope

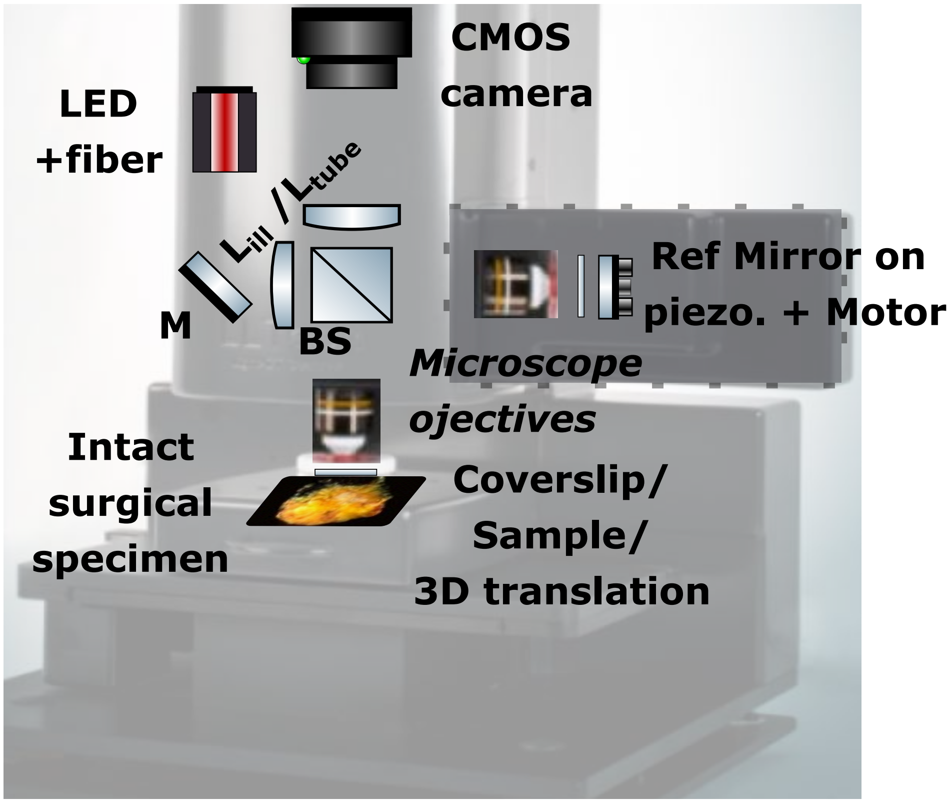

Figure 1: Rough optical design behind the LightCT microscope. The system is composed of a spatially and temporally incoherent light source (typically LED) that illuminates a Linnik interferometer via a turning mirror (M) and a lens (L). The interferometer is composed of a beamsplitter (BS) that splits the illumination light between a sample and a reference arm. The sample arm contains a 10X, 0.3 NA microscope objective, the intact surgical specimen gently pressed against a 1mm thick coverslip, as well as a 3D translation stage. The reference arm contains the same objective and the same glass coverslip but images a simple mirror instead of the sample. The reference mirror is mounted on a piezoelectric transducer to rapidly modulate the optical path difference and achieve 4 phases imaging to recover the amplitude of the interference term. The entire reference arm is mounted onto a motorized translation to adjust the coherence volume (i.e; the imaged plane in the sample). Light beams backscattered from both arms are recombined by the BS, and the image of the sample is made onto a CMOS camera thanks to a tube lens. The system design is superimposed on a picture of the actual system.

Description of features used by histopathologists to perform the diagnosis, with standard histology, static and dynamic FF-OCT.

In order to perform the manual classification of static and dynamic FF-OCT images, and in general, the pathologists were looking for a few morphological features, including:

- For healthy samples, pathologists were looking for normal lobules (with normal size cells and normal cell organization), normal ducts (cells only on the walls but not in the opening), collagen fiber organization, the presence and density of blood vessels, adipocytes, immune cells, and muscle.

- For abnormal samples, a search for abnormal lobules (with abnormal size cells and/or abnormal cell organization), invaded ducts (with cells present in the opening), cell invasion in collagen is made. Other features such as collagen disorganization, excess of collagen, the presence of unidentified cell clusters, or clusters.

The easiest features to assess for the pathologists in the FFOCT and D-FF-OCT images were the lobules and ducts aspect (See figure 3), the presence of cell clusters, the collagen organization. The most difficult features were the identification of immune cells, because they can be difficult to differentiate from other cells (although they should have a much larger and faster -resulting in a white aspect in the color D-FF-OCT image), the necrosis because it can take various apparences, and because by definition necrotic structures don't appear in dynamic FF-OCT, and the cell invasion in collagen because of the low contrast of these cells.

Detailed description of false positive and false negative cases.

Regarding the manual classification using small ROIs imaged with dynamic FFOCT, there were 4 and 3 false-negative cases, respectively, for the 2 pathologists, including 3 in common. Among the false-negative cases, 1 was not included in the dataset tested by the deep learning algorithm (because data annotation was too complex), 1 was also misclassified by the deep learning algorithm, 2 were successfully classified by the algorithm.

Description of false negatives.

We found that the main reason for false-negative cases was missed isolated cancer cells (figure 2), which were weakly contrasted (especially in those cases) with surrounding connective tissue and were often localized in a small portion of the ROIs. We postulate that our automated algorithms are more efficient to detect such cells because they use the three independent RGB channels. As an example, although this is difficult to see isolated cancer cells using the color D-FF-OCT image (figure 2 A), it is possible to enhance the contrast of these cells using image processing tools (figure 2 C), e.g. by subtracting the green channel to the blue channel. Because the two algorithms use embedded image processing tools, we imagine that they are quite efficient in detecting single cells even above a large background of fibers. Besides, the feature engineering algorithm computes the number of cells outside clusters that is dedicated to finding such isolated cancer cells.

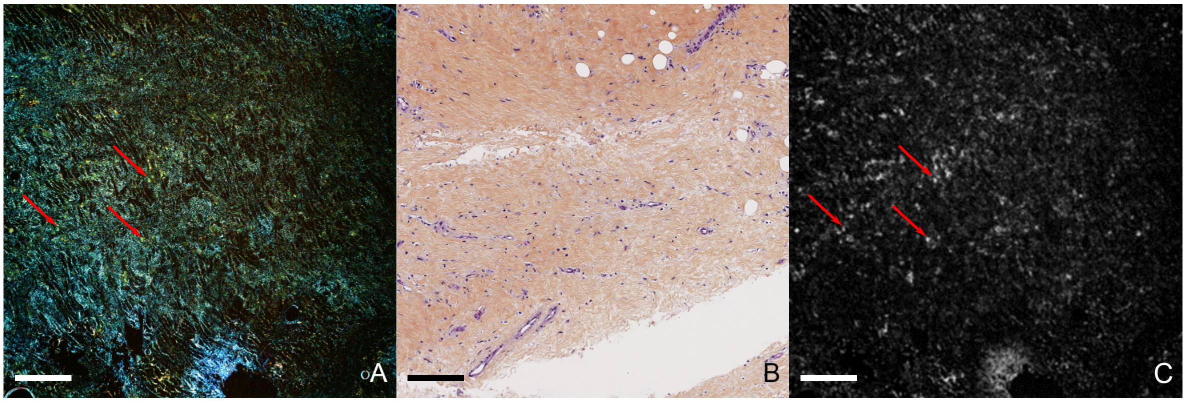

Figure 2: **Illustration of a shared false negative case**, misclassified by P1, P2 and CNN algorithm. (A-B) Invasive isolated cancer cells (red arrows) in invasive lobular carcinoma are less contrasted with collagen fibers on Dynamic FF-OCT image (A) than on histology image (B) and were probably missed by the pathologist. (C) Dynamic FF-OCT image where the blue channel was subtracted to the green channel, suggesting that isolated cancer cells can be more easily detectable with the adequate image processing tools. Scale bars represent 250 μm.

Description of false positives.

There were 1 and 3 false-positive cases respectively, for the 2 pathologists, including 1 in common. Among the false-positive cases, 2 were also misclassified by the deep learning algorithm and 1 was successfully classified. As shown in figure 3, the main reasons for false-positive cases were surprisingly highly-contrasted normal cells mistaken as cancer cells or abnormal benign lobules or ducts mistaken as cancerous. Although we do not have a strong explanation for the highly contrasted cells (figure 3A), the entire dynamic images seem brighter and "yellower" (indicating faster dynamics) compared to other samples. It may relate to an additional fast mechanical noise during the data acquisition, and we could imagine discarding some images if their average color histogram is too different as a typical histogram. Regarding the abnormal benign lobules or ducts, some context is missing to provide an accurate manual diagnosis to verify that the surrounding tissue is normal. The automated algorithms probably lack some data and annotation, including several cases of abnormal but benign regions. In this study, only two healthy cases had such abnormal regions. As an example, one important feature of the feature engineering approach is the eccentricity of the regions of high cell density as the ones shown in figure 3C. Because the ducts are abnormal and elongated, the eccentricity is largely below 1. Because this was the only case with abnormal ducts, whereas other non tumoral healthy ducts are circular, it results in a high probability of being a tumoral ROI. Nonetheless, because most of the other ducts were normal, the proportion of normal ROI was above 0.5 leading to a good classification at the sample scale. With more similar abnormal but healthy ducts in the training set, the predictive ability of the eccentricity of regions of high cell density alone would be mitigated, which should improve the diagnosis ability.

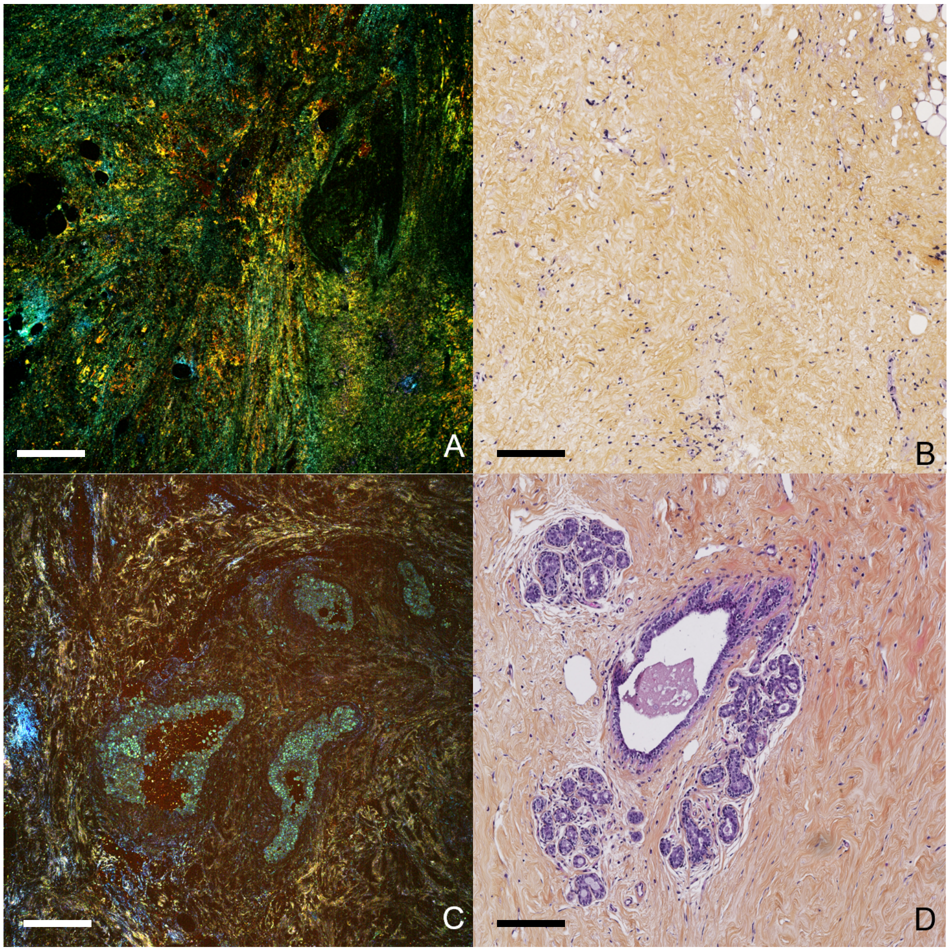

Figure 3: **Illustration of a shared false positive case.**(A-B) misclassified by P2 and deep learning algorithm, (C-D) misclassified by P1, P2 and deep learning algorithm. (A-B) False positivity of normal connective tissue attributed to DCI-detected cells of high intensity, probably mistaken as invasive cancer cells instead of fibroblasts. (C-D) Dilated duct mistaken as invaded duct. Scale bars represent 250 μm.

Detailled description of Ilastik and Matlab cell and fiber segmentation.

The first step of the engineered features analysis on breast samples was to perform cell and fiber segmentation in the images, using *iLastik*. *iLastik* is a relatively intuitive machine-learning tool based on random forest classifiers. The labels are manually drawn on the training images in a user interface. Each pixel neighborhood is characterized by a set of generic nonlinear spatial transformations calculated by the software and applied to each channel (R, G, or B) of the D-FF-OCT image. The same transformations were applied to the greyscale FF-OCT images. The following image transformations empirically gave the best contrast in our case:

1. **Gaussian smoothing** with a increasing standard deviation of 0.3, 0.7, 1.0, 1.6, 3.5, 5.0.

2. **Laplacian calculation after Gaussian smoothing** with a standard deviation of either 0.7, 1.0, 1.6, 3.5, or 5.0. This computes the edge of the objects.

3. **Difference of Gaussians** with a standard deviation of either 0.7, 1.0, 1.6, 3.5, or 5.0. It also computes the edge of the objects, by subtracting two images after Gaussian

smoothing with almost similar standard deviations and is supposed to approximate a derivative.

4. **Hessian of Gaussian Eigenvalues** with a standard deviation of either 0.7, 1.0, 1.6, 3.5, 5.0. It computes the local texture of the image by calculating the determinant of the Hessian matrix.

In total, for all the D-FF-OCT images in the dataset, 21 transformed images are calculated per color, hence 63 grayscale images, plus 21 images for FF-OCT images. For both FF-OCT and D-FF-OCT datasets, the training step was performed on 8 images (5 from cancerous samples and3 from healthy samples). For D-FF-OCT, we defined three classes: *Cells, Between Cells, Not Cells (Fibers, Noise, Fat, etc...)*, out of which only the *Cell* class was used. A few pixels corresponding to each class are drawn manually, as shown in figure 4A. The best practice is to draw a small number of pixels to minimize constraints, and to draw pixels at the interface between classes (meaningful pixels). The classifier (figure 4B) should be created as soon as a few pixels of each class are annotated (typically a few cells, and a few spaces between cells in the first image), and then finely tuned by manually changing the class of misclassified pixels in all 8 images. The whole training procedure can last for around 30 minutes, and is not highly accurate since a few cells and cell contours are missed. High accuracy is not necessary though given than the expected outcome is not a real metric of cell sizes or shapes, but how these parameters compare from one sample to another.
Segmentation of FF-OCT was performed similarly, although we used 4 classes: *Fibers, Around Fibers, Cells (and Noise), Fat (and holes/ slicing artifacts)*. The *Around Fibers* class is used to be able to segment individual fibers, and not only the regions of high fiber density. The *Cells* class contains most of what is not fibers, including extracellular matrix and cells. We later split this data into bright pixels (50% brightest pixels), most likely forming the extracellular matrix, darker pixels (between 25% and 50% brightest pixels)being part of cells, and we excluded the darkest pixels (¡25%). The *Fat* class segments dark pixels in circular regions, as observed in fat regions of the breast (Figure 4A1 and 4A2 of the main manuscript in blue). Unfortunately, it also finds a few holes in the sample, caused by slicing, folding artifacts, or bubbles.
In total after these learning steps, each FF-OCT and D-FF-OCT image can be segmented in about 10 seconds.

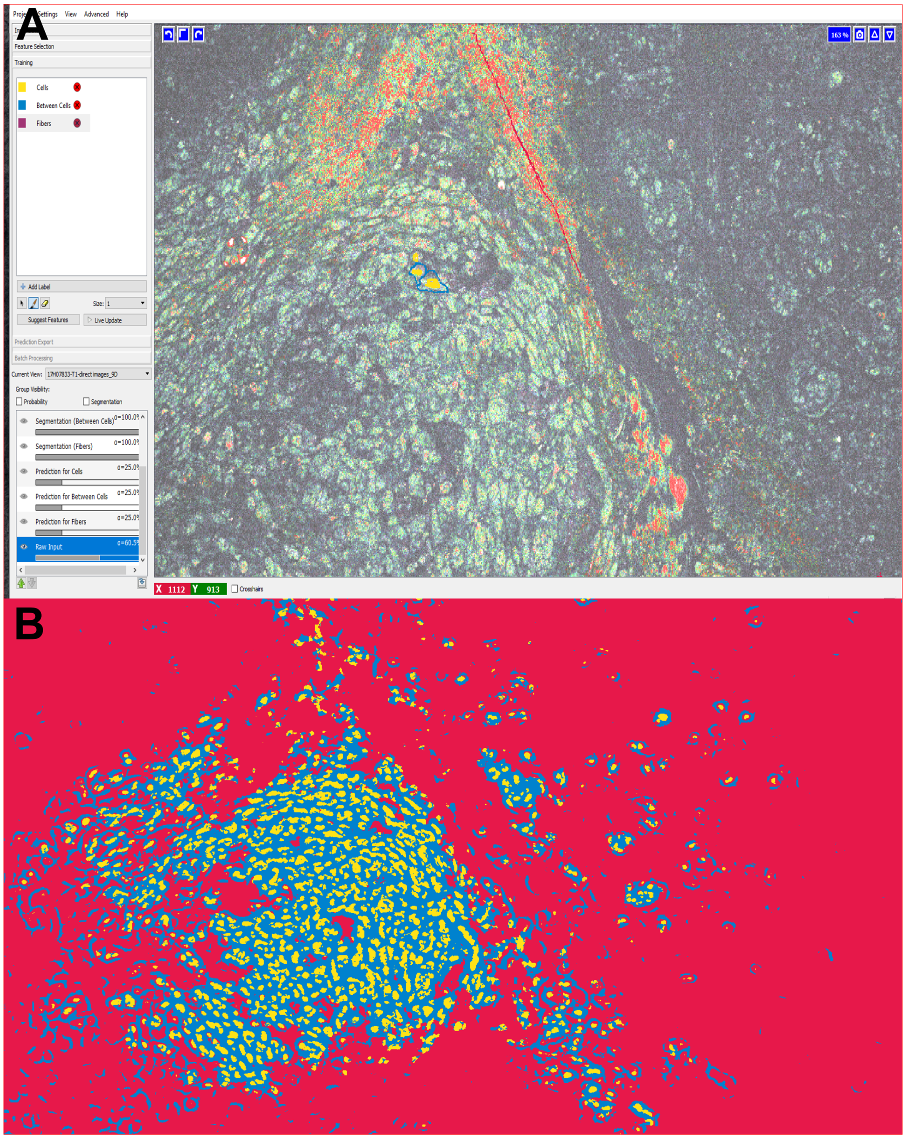

Figure 4: Machine learning based segmentation with iLastik on D-FF-OCT images. Panel A shows the user interface of iLastik, and illustrates the learning procedure. Pixels corresponding to each class of interest are manually drawn (opaque pixels). To start with initial prediction, a small number of pixels in the first image were drawn, and then the prediction is refined step by step by correcting misclassified pixels. Panel B shows the segmentation result after the learning process. For D-FF-OCT images, only the cell segmentation is used (class 1 - in yellow here), but the others two classes were used to segment the cells with higher precision.

After an initial segmentation step by *iLastik*, the binary maps are imported in *Matlab*, and each connected set of pixels is individually detected by the function *regionprops*. Using D-FF-OCT segmented images, objects corresponding to single cells are extracted and, given

their area is significant, their morphological and scattering parameters are extracted (See figure 5 A). Using FF-OCT segmented images, objects corresponding to fibers are extracted (see figure 5 B), as well as the regions corresponding to the sample stroma.

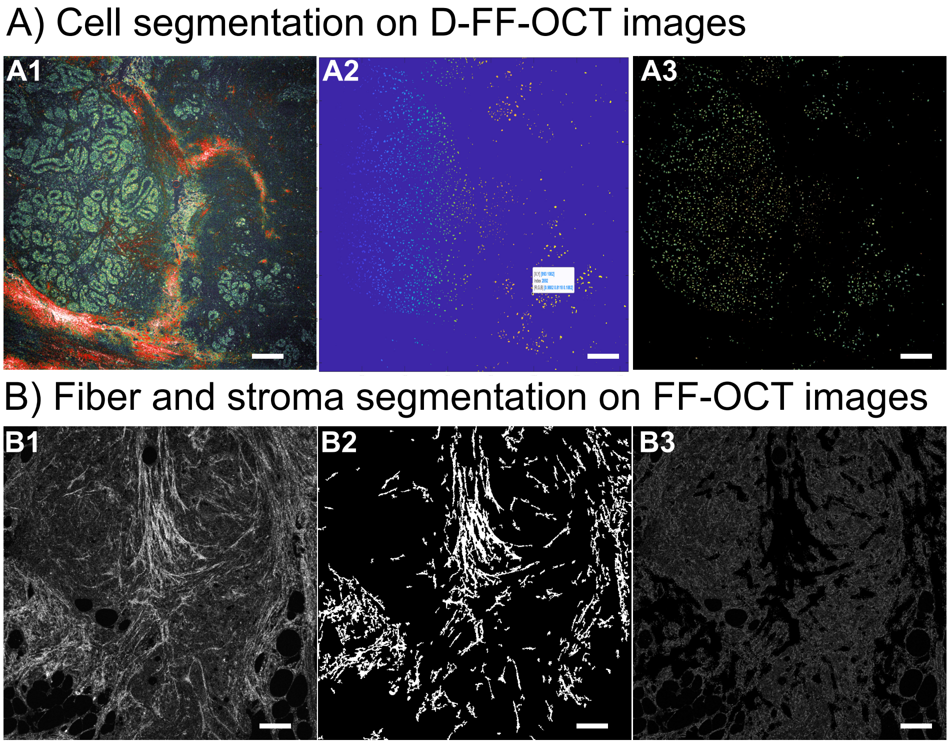

Figure 5: Cell and fiber segmentation output. Panel A shows the cell segmentation using iLastik and Matlab. From original D-FF-OCT image (A1), after iLastik segmentation, the first class (cells) is selected, and separated in independent regions with Matlab (A2). Here, the increasing color from blue to red gives the number of the identified region (from 1 to 3951 here).Each region of interest (ROI) can be analyzed independently, and only the regions of area above 20 pixels are kept and considered as cells. The obtained mask image is multiplied by the D-FF-OCT image to obtain panel A3. Panel B shows the fiber and cell segmentation results on FF-OCT image. The original image (B1) is processed by iLastik and classified between fibers, pixels between fibers, and cells. The first and third classes are extracted and filtered using Matlab to obtain fibers (B2) and stroma (B3). Scale bars are 150 μm

Definition of the 44 features used in the feature engineering approach.

The table below describes the 44 features used for the SVM analysis in the feature engineering approach. For some features (e.g. diameter of segmented cells) where several observations are available in each ROI (e.g. one diameter per cell- leading sometimes to hundreds of values associated to each ROI), we only kept the average value and the standard deviation, hence defining 2 features. For example, the first line of the table means that the first feature is the average cell diameter in each ROI, and the second feature is the standard deviation of the cell diameter distribution obtained in each ROI. The most contributing features (see feature reduction) are underlined in green. For example, the first line should be understood so that the second feature (standard deviation of cell diameter) only has a high contribution to the SVM segmentation. It can be interpreted as the average segmented cell diameter is not statistically different in healthy versus tumoral ROIs. In contrast, it is likely that some tumoral ROIs have

a larger distribution of cell diameter distribution (e.g. with healthy cells of regular diameter and some tumoral cells of increased diameter).

Table 1: Calculated features from image analysis of static and dynamic FF-OCT images. For some features (e.g. cell diameter), a distribution is first calculated, but the extracted features are the mean and standard deviation (STD) of the distribution. Features with green underline correspond to the features kept by the algorithm after feature reduction.

Number	Type of feature	Description of the feature	Additional comment
1 & 2	Cell features from D-FF-OCT	Diameter of segmented cells (Mean and STD)	Measure Cell size
3 & 4	Cell features from D-FF-OCT	Eccentricity of segmented cells (Mean and STD)	Measure Cell shape
5	Cell feature from D-FF-OCT	Total cell density	$Density = \frac{N_{seg.cells}}{N_{pixels}}$
6 & 7	Cell features from D-FF-OCT	Average intensity within each segmented cell (Mean and STD)	Scattering is expected to increase in cancerous cells
8	Cell feature from D-FF-OCT	Mean intensity of the red channel of all pixels classified of the *cell* class	Fast dynamics : Cancerous cells are expected to have increased metabolic activity, hence faster and stronger fluctuations.
9	Cell feature from D-FF-OCT	Mean intensity of the green channel of all pixels classified of the *cell* class	Intermediate dynamics
10	Cell feature from D-FF-OCT	Mean intensity of the blue channel of all pixels classified of the *cell* class	Slow dynamics
11 & 12	Mesoscale features from D-FF-OCT	Local cell density in regions of high cell density (Mean and STD)	High cell density : At least 5% of the surrounding pixels belong to the *cell* class.
13& 14	Mesoscale features from D-FF-OCT	Area of regions of high cell density (Mean and STD)	In healthy samples, the lobules are high cell density regions but they are well organized and small.
15& 16	Mesoscale features from D-FF-OCT	Eccentricity of regions of high cell density (Mean and STD)	

Continued on next page

Table 1: Calculated features from image analysis of static and dynamic FF-OCT images. For some features (e.g. cell diameter), a distribution is first calculated, but the extracted features are the mean and standard deviation (STD) of the distribution. Features with green underline correspond to the features kept by the algorithm after feature reduction. (Continued)

Number	Type of feature	Description of the feature	Additional comment
17	Cell feature from D-FF-OCT	Number of cells measured outside regions of high cell density	Migrating cells should not be found in healthy samples.
18-21	Cell features from D-FF-OCT	Spatial STD of intensity and STD normalized by intensity) within each segmented cell (2 means and 2 STDs)	Measure a the intensity heterogeneity (STD(I) and STD(I)/I) inside each cell.
22 & 23	Fiber features from FF-OCT	Diameter of segmented fibers (Mean and STD)	Measure collagen fiber size
24 & 25	Fiber features from FF-OCT	Eccentricity of segmented fibers (Mean and STD)	Measure collagen fiber shape
26 & 27	Fiber features from FF-OCT	Angular distribution of segmented fibers (Mean and STD)	Measure collagen fiber organization, possibly altered in cancerous samples.
28	Fiber features from FF-OCT	Density of segmented fibers	Possibly increasing in cancerous samples
29 & 30	Fiber features from FF-OCT	Mean intensity of each segmented fiber (Mean and STD)	Scattering of the extracellular matrix is possibly increasing in cancerous samples.
31 & 32	Cell features from FF-OCT	Mean and STD of intensity of all pixels that belongs to the *cell* class.	Scattering of cancerous cells are possibly increasing.
33	Cell features from FF-OCT	Mean intensity of the 25% brightest pixels that belongs to the *cell* class.	
34	Mesoscale features from FF-OCT	Number of pixels belonging to the regions of high fiber density	
35 & 36	Mesoscale features from FF-OCT	Average fiber density within each region of high fiber density (Mean and STD)	
37 & 38	Mesoscale features from FF-OCT	Area of each region of high fiber density (Mean and STD)	

Continued on next page

Table 1: Calculated features from image analysis of static and dynamic FF-OCT images. For some features (e.g. cell diameter), a distribution is first calculated, but the extracted features are the mean and standard deviation (STD) of the distribution. Features with green underline correspond to the features kept by the algorithm after feature reduction. (Continued)

Number	Type of feature	Description of the feature	Additional comment
39 & 40	Mesoscale features from FF-OCT	Eccentricity of each region of high fiber density (Mean and STD)	
41	Mesoscale features from FF-OCT	Number of regions of high fiber density	
42	Fat features from FF-OCT	Average intensity of all pixels belonging to the *Fat* class	
43	Fat features from FF-OCT	hl Number of pixels belonging to the *Fat* class	
44	Cell features from FF-OCT	Mean intensity of the 25%-50% least bright pixels that belongs to the *cell* class.	

Influence of the penalization coefficient used in the FE and CNN approaches

For both the feature engineering and CNN approaches, we used different weight, or penalization coefficients, in the training step, so that to compensate for class imbalance, and to give more weight to the healthy ROIs.

In general, if the data imbalance is not compensated for, the algorithms tend to favor the class with the more examples, since it only maximizes the total accuracy. In this case, it would result in algorithms overpredicting tumoral ROIs, hence a minimal change in accuracy but a loss of specificity.

In order to illustrate this effect with the feature engineering approach, we trained and tested several linear SVM with a varying penalization coefficient from -5 to 5. A penalization coefficient of -5 means that false negative are penalized with a weight of 5, while a penalization coefficient of 5 means that false positives are penalized. As a reminder, in the main study, the penalization coefficient is chosen to be 3 corresponding roughly to the ratio between the number of tumoral versus healthy ROIs.

Figure 6 shows the accuracy, sensitivity, and specificity obtained for these different penalization coefficients. As expected, the accuracy is roughly constant. However, the specificity increases sharply with the penalization coefficient, which means that the model tends to overpredict tumoral ROIs when false negatives are penalized or when the data is imbalanced (Penalization coefficient of 1). In contrast, the sensitivity starts decreasing when the penalization coefficient is too large, *i.e.* when the false positives are penalized too strongly.

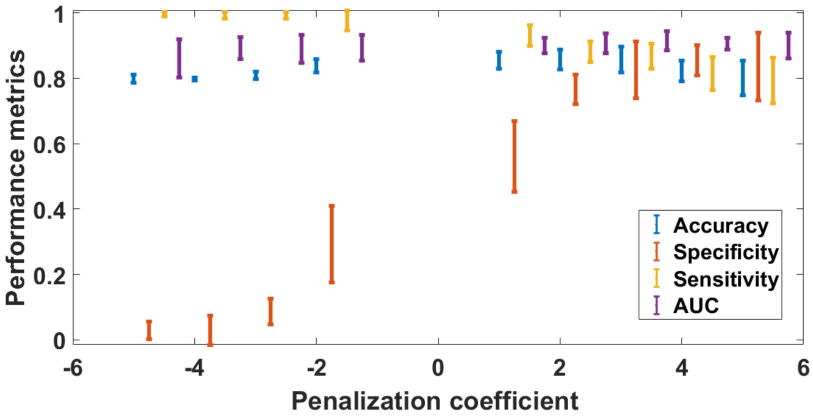

Figure 6: **Prediction scores of linear SVM models trained with varying penalization coefficient.** Accuracy (blue), sensitivity (yellow), specificity (orange), and AUC (purple) are plotted versus the penalization coefficient used when training the SVM model. Negative coefficient means that false negative are penalized, while positive coefficients correspond to penalizing false positives.

Figure 7 shows the equivalent of figure 4D, obtained for different penalization coefficients. It shows the translation of this loss in specificity and sensitivity at the ROI level caused by class imbalance, into the tissue scale. As mentioned above, for negative coefficients, when false negatives are penalized, the SVM tends to predict only tumoral ROIs to maximize the accuracy. When merging all ROIs, it translates into all samples being predicted as tumoral samples. In contrast, as the false positives become more and more penalized, the global ratio of healthy vs tumoral ROIs increases. It therefore becomes possible to differentiate healthy samples (blue dots) that exhibit higher ratios. The accuracy at the tissue level (*i.e.* the separability between healthy and tumoral samples) is found maximal with a penalization coefficient of 3, that corresponds to restoring class balance, and as chosen in the main study. However, the models would still be effective for penalization coefficients of 2, or 4, around the optimal value.

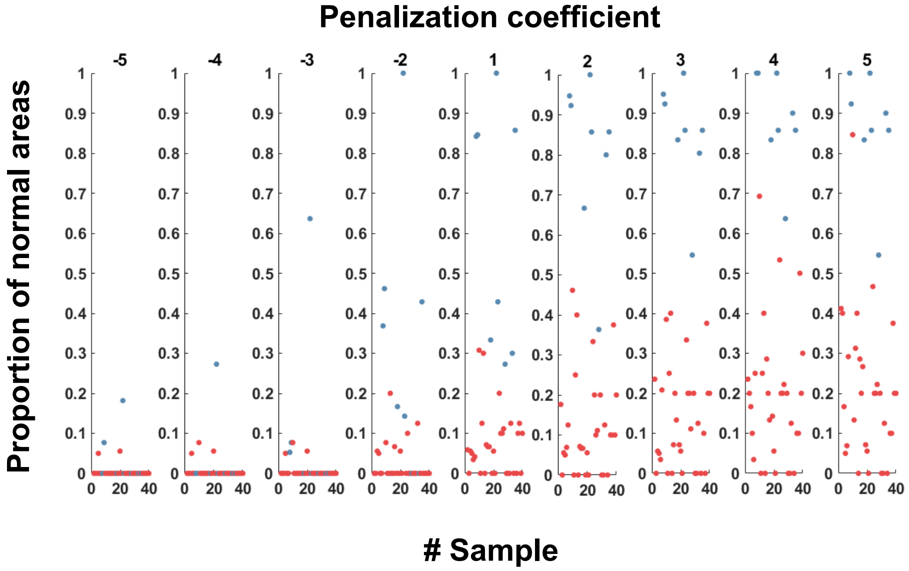

Figure 7: **Proportion of normal areas found for each healthy (blue) and cancerous sample (red) obtained for SVM models trained with varying penalization coefficients.**

ROC curves obtained with the linear SVM model.

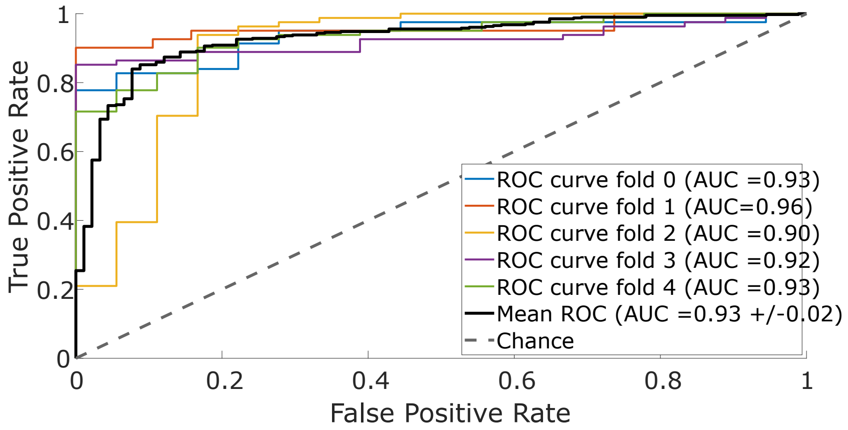

Figure 8: AUC Curves linear SVM
